# A simple modified Bentall technique for surgical reconstruction of the aortic root – short and long term outcomes

**DOI:** 10.1186/s13019-015-0336-4

**Published:** 2015-10-26

**Authors:** Pouya Nezafati, Ali Shomali, Mohammad Hassan Nezafati

**Affiliations:** 1 Department of Cardiac Surgery, Imam Reza Hospital, Mashhad University of Medical Sciences, P.O. Box: 9137913316, Mashhad, Iran; 2 Javad Al Aemeh Hospital, Mashhad, Iran

**Keywords:** Aortic root replacement, Modification, Aortic surgery, Aortic valve

## Abstract

**Background:**

Since the first introduction of the Bentall technique, several modifications have been proposed to improve patient outcomes and decrease intra- and post-operative complications. We describe a simplified modification of the technique that tries to lessen the intra-operative time, improve homeostasis and miminize early and late complications. Our experience with the technique and short- and long-term patient outcomes are reported.

**Methods:**

From August 1996 to October 2013, 110 consecutive patients underwent this modified technique. The procedure used Dacron composite graft with a mechanical valve (St. Jude Medical®) for aortic root replacement. To avoid intra-operative complications, no mobilization of coronary ostia was done. Additionally, the tubular aorta was kept minimally unchanged.

**Results:**

Total bleeding after the operation was 450 ± 105 mL. The mean duration of intensive care unit and hospital stay were 2 ± 1 and 5 ± 2 days, respectively. Sixty-six patients (60 %) were discharged from the surgical intensive care unit on the first postoperative day, 34 patients (30.9 %) were discharged on the second day and ten patients (9.1 %) needed more time to stay in the intensive care unit due to haemodynamic or respiratory problems. At 5-years follow up, survival rate was 97 %. In the three deceased patients, causes of death were mediastinitis, sepsis and myocardial infarction. No operation-related complications such as anticoagulant-related hemorrhage, valve or graft thrombosis, or coronary pseudoaneurysm were occurred during follow-up.

**Conclusions:**

The proposed modification of the Bentall technique seems to minimize late intra-operative blood loss, improves homeostasis, shortens the operation time and is associated with excellent long-term outcomes in patients undergoing composite graft replacement of the aortic root.

## Background

Aortic root surgery with a valved composite graft is used to modify thoracic aortic abnormalities in the aortic root [1]. In 1968, Bentall and De Bono were the first to describe the surgical procedure for the reconstruction of the aortic root with a valved composite graft [2]. For years, this technique became the practice standard for surgical treatment of dysfunctions of the aortic valve, root and ascending aorta [3–5]. In the original method, late intra-operative bleeding was controlled by means of circumferential suture lines on the coronary peri-ostium areas and an overall aortic wraparound [2]. In the initial series, the postoperative complications like coronary detachment, formation of false aneuryms and need for re-operation in the Bentall operation were very high [2]. Since then, with increased experience of the surgeon and technical improvements such as pre-clotting of grafts with albumin, enhancements in pump oxygenator systems, and accurate heparin adjustments the rate of complications has significantly dropped [6]. Several modified techniques have also been developed to further reduce the rate of complications and improve patient putcomes [4, 7–9].

Currently, the modified Bentall techniques that incorporate coronary button mobilization have become the precodure of choice in many centers around the world [10–12].

The Bentall technique involves direct reimplantation of the coronary arteries, whereas the modified techniques require formation of ostial “buttons” that are then attached to the graft. Despite significant improvements with modified techniques, intra-operative blood loss and post-operative complications remain a major obstacle. Therefore, the present study aimed to evaluate a novel modification that tries to lessen intra-operative time, improve homeostasis and miminize early and late complications. Our experience with this method in a sample of 110 patients are described and long-term outcomes are provided.

## Methods

### Patients

From August 1996 to October 2013, 110 consecutive patients [84 men (76.4 %) and 26 women (23.6 %)] with a mean age of 48.0 ± 7.4 years (range: 19–78 years) underwent composite graft replacement of ascending aorta at Javad Al-Aemeh hospital, Mashad, Iran. Patients with dissection of the aortic arch (excluding the innominate artery), thoracic aorta or abdomino-thoracic aorta not included. These pathologies were operated by other methods including hybrid procedures in some cases. Patients with ascending aorta (DeBaky type II) including the innominate artery dissection were included in this study. Patient medical records, case notes and follow up documents were reviewed and information regarding patient demographics, indication for surgery, co-morbid medical condition, details of pre-, intra- and post-operative procedures, early and late complications and outcomes were retrieved and recorded in pre-designed sheets. All procedures dealing with human subjects were conducted in accordance with the guidelines laid down by the Helsinki declaration. After explaining the nature of the surgery and its possible complications, written informed consent was obtained from all patients. Ethics committee of the Mashhad University of Medical Sciences also approved the study.

### Surgical technique

**Fig. 1:**
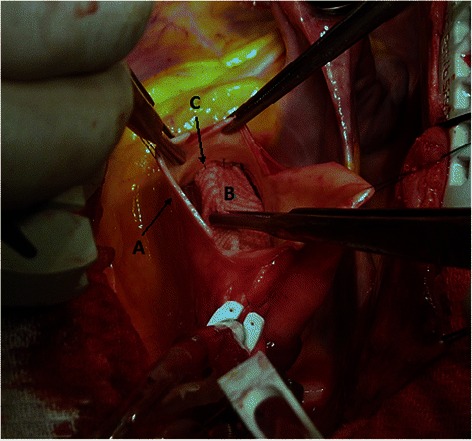
Dacron composite graft placed inside the aorta: **a** wall of the excised aorta, **b** Dacron composite graft, **c** Sutures of the right coronary ostium to the graft

**Fig. 2:**
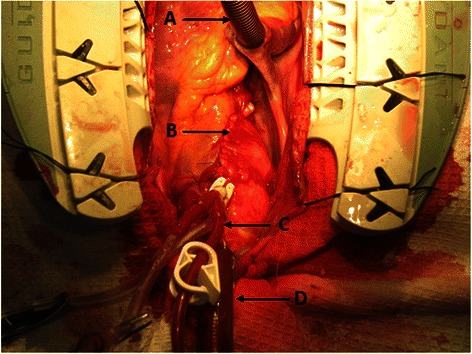
Aortic wall closed and covers the dacron composite graft: **a** Single venous Cannula, **b** Sutures of the aortic wall, **c** cardioplegia cannula, **d** Arterial cannula

After inducing general anesthesia and confirmation of hemodynamic stability, a median sternotomy was performed. In cases where the aneurysm did not involve the distal portion of the ascending aorta or proximal portion of the aortic arch, these sites were used for arterial cannulation. In cases with extended disease, however, the right femoral artery was chosen as the site of entry. Venous cannulation was achieved by a single two-stage cannula or bicaval cannulation strategy. Upon reaching an activated clotting time of >480 s cardiopulmonary bypass was instituted. Patients with dissection of the ascending aorta including dissection of the innominate artery were placed under hypothermic circulatory arrest. Also, in cases with ascending aortic dissection presenting up very close to the innominate artery but not involving the innominate artery, the aortic clamp was placed at the base of the innominate artery which resulted in lowering the blood flow through the innominate artery, so the temperature was lowered to 25–28°. In other cases which the ascending aorta was clamped just below the innominate artery, the operations were performed under hypothermia at 32° rectal temperature. The heart was fibrillated using local ice cooling, followed immediately by cross-clamping of the aorta near the origin of the innominate artery. A longitudinal aortotomy below the cross-clamp was made. For cardioprotection, the cardioplegic solution was then administered in an antegrade manner into the coronary ostia. Repeat doses were infused every 20–30 min throughout the procedure. Cold saline was administered topically to augment myocardial cooling. To protect the brain tissue, antegrade cerebral perfusion was instituted at 10 ml/kg/min. To avoid damaging the right coronary ostia, the incision was extended towards the non-coronary sinus. No mobilization or dissection of ascending aorta from surrounding tissues was necessary. A composite graft consisting of a St. Jude valve (St. Jude Medical, St. Paul, Minnesota) and a Dacron tube graft (Boston Scientific Corp., Wayne, New Jersey), was used to replace the diseased portion of the aorta. The size of the Dacron tube graft was chosen to be equal to the outer diameter of the mechanical valve’s sewing cuff. The most frequent graft size used was 24 mm (45 %) and the least common graft size was 28 mm (3.2 %). Running sutures with 4-0 propylene were used to attach the tube to the sewing cuff. The aortic cusps were removed and the annulus was debrided in the presence of calcification. An adequate portion of the aneurysmal aortic wall was excised just enough to place the Dacron tube graft inside with no free space left between walls of the tube graft and aorta. After sizing of the aortic annulus, a series of pledgeted mattress sutures were placed. After completing the suture line, the coronary buttons in the tube graft were created. Round openings of 2 to 3 times larger than the diameter of coronary ostia were made in the graft and remnants of the arterial walls around the coronary arteries were sutured to the graft in an end-to-side fashion with continuous 5-0 polypropylene sutures (Fig. 1). No additional material was used to reinforce the anastomosis. Care was taken to implant coronary arteries directly without dissecting the coronary ostia or making tension in any direction, in order to prevent kinking and/or bleeding when cardiopulmonary bypass ceases and heart beating is resumed. In the last stage, the distal end of the conduit was anastomosed to the distal ascending aorta with continuous 3-0 polypropylene sutures. Blood cardioplegic was injected into the graft under pressure before performance of the distal anastomosis to prevent oozing and bleeding from coronary ostia. Finally, after confirmation of adequate hemostasis, the remaining aortic wall was used to cover the Dacron graft (Fig. 2), without plicating the aortic wall, since covering accelerates hemostasis and coagulation in suture lines.

### Post-operative management

All patients received intravenous heparin until the ablation of epicardial electrodes, followed by oral anticoagulation using fluindione (a warfarin-like antivitamin K drug, with a half-life of 31 h). International normalised ratio was targeted between 2 and 3, as recommended by the mechanical valve manufacturer. Administration of aspirin in combination with oral anticoagulants was left to the discretion of the cardiologist.

### Statistical analysis

Statistical analyses were carried out using the the Statistical Package for the Social Sciences (SPSS) version 16.0 (SPSS Inc., Chicago, Illinois). At first, quantitative data were assessed using Kolmogrov-Smirnov tests for normality. Data were expressed as means ± SD for parameters with a normal distribution or median and interquartile range for non-normally distributed data. Group comparisons were performed using independent *T*-test for variables with normal distributions, or Mann–Whitney *U* test for variables with non-normal distributions. A two-sided P value < 0.05 was considered statistically significant. Linearised rates, calculated as the number of events divided by total follow-up years and then multiplied by 100 to convert to the percentage per patient-year, were used to describe late valve-related complications.

## Results and discussion

Baseline characteristics of patients are presented in Table 1. Indications for surgery encompassed annuloaortic dissection in 74 patients (67 %), chronic dissection and acute dissection of the ascending aorta in 23 (21 %) and 13 (12 %), respectively. Thirty patients (27.3 %) had prevrious cardiac surgery. Ten cases had undergone aortic valve replacement (6 isolated, 1 combined with replacement of the supracoronary aorta and 3 with coronary artery bypass grafting [CABG]). Among the 30 patients who had a previous cardiac surgery, cardiopulmonary bypass time was significantly longer in comparison with the first time operated patients (192 ± 34 vs 144 ± 48 min; *p* = 0.02).

**Table 1:** Baseline characteristics of patients undergoing composite valve graft replacement of the aorta

Sex (Male/Female)	84/26 (67.9/32.1)
Age (years) ± SD	48.0 ± 7.4
Pre-existing conditions n, (%)	
Hypertension	83 (75.5)
Diabetes Mellitus	11 (10.0)
Dyslipidemia	31 (28.2)
Coronary artery disease	35 (31.9)
Chronic obstructive pulmonary disease	22 (20.0)
Chronic kidney disease	4 (3.6)
Marfan syndrome	6 (5.5)
Smoking n, (%)	48 (43.6)
Bicuspid aortic valve n, (%)	13 (11.8)
Previous Cardiac Surgery n, (%)	30 (27.3)
Aortic regurgitation	97 (88.2)
Aortic regurgitation + aortic stenosis	6 (5.5)

A description of intra-operative characteristics are presented in Table 2. Following operation, one patient developed low-cardiac-output syndrome. This was successfully managed by the use of intra aortic ballon pumping and the infusion of inotropic agents. The mean duration of Intensive Care Unit (ICU) stay was 2 ± 1 days. Sixty-six patients (60 %) were discharged from the surgical ICU on the first postoperative day, 34 (31 %) were discharged on the second day; ICU stay in the remaining 10 patients (9 %) was more that 2 days. The mean duration of hospital stay was 5 ± 2 days. Total circulatory arrest was done on seven patients; Also, temperature was lowered to 25–28° in 59 patients and 44 operations were performed under hypothermia at 32° rectal temperature.

**Table 2:** Intraoperative data in patients undergoing composite valve graft replacement of the aorta

Operation time (min)	388 ± 86
Cardiopulmonary bypass time (min)	144 ± 48
Total circulatory arrest time (min)	22 ± 18
Aortic Cross Clamp Time (min)	68 ± 37
Low cardiac output syndrome n, (%)	1 (1.1)
Additional procedures n, (%)	20 (18.2)
Composite valve size (mm)	23.4 ± 3.6

### Post-operative bleeding

The average post-operative blood loss was 450 ± 105 mL (326 ± 95 mL in the first post-operative day and 130 ± 64 mL in the second day). Twelve patients (11 %) experienced blood losses of 1000 mL or more; and in four cases chest re-exploration for bleeding was required. The source of bleeding in three patients was hemorrhage from the distal tube graft anastomosis, and in the other, oozing from the proximal tube graft anastomosis was identified. In all four cases, bleeding was controlled by enforcing the anastomosis site by means of additional sutures. In the remaining eight patients with blood losses of more than 1000 mL, conservative medical management in the ICU proved to be sufficient. The average number of blood unit transfusions was 2.5 (range: 1 to 4 units). However, patients who experienced kidney injuries and respiratory complications received more units of blood (5.0 and 3.5 units, respectively).

### Complications

Acute kidney injury occurred in three patients and resolved with medical management. Neurologic sequelae was observed in four patients; one had transient ischemic attack and in the other three ischemia progressed to cerebrovascular accident. In all four, brain computed tomography failed to identify embolic causes and diffuse hypoperfusion was considered as the underlying cause. Four patients experienced respiratory complications requiring prolonged ventilation. No cases of endocarditis, pericardial effusion, or gastrointestinal bleeding were diagnosed.

### Short and long-term postoperative follow-up

After discharging from hospital, patients were visited 7-days, 30-days after discharge and annually thereafter. The follow-up schedule consisted of routine laboratory tests, chest radiography and echocardiography. During the follow-up period three patients died giving rise to an overall survival rate of 97 %.

One patient died 3 months after surgery due to mediastinitis. Another patient died due to sepsis 34 months after the procedure. The patient had rheumatoid arthritis and had been taking predonisolone for the past 15 years. An infection of unknown origin caused sepsis and he died due to multi-organ failure. Another patient died 42 moths after the surgery due to myocardial infarction.

The current study’s modified Bentall technique believes to be simplified, thus resulting in shortening of the operation time. Simplification consists of no dissection or mobilization of coronary ostia. As a dilated root is frequently associated with cephaled migration of the coronary ostia, approximation of the aortic wall to the graft buttons should be made directly and in front of the ostia by pre-marking the place in which the buttons should be placed. Although dissection of the ostia allows a better exposure and visualization of the coronary anastomosis, this could cause collateral damage, torsion of coronary ostia, pseudoaneurysms and coronary stenosis. So, the injury to the coronary ostia and the proximal coronary artery could result greater post-operative complications. However, as in this technique a great caution and patient is required to perfomre the suture line without dissection of the coronary ostia to minimize the mentioned complications. It is to note that since we do not excise the aneurysmal wall of the sinus of valsalva and sino-tubular junction, there is enough wall left that enables a proper, tensionless and well-fixed end to side suture line. So, as no tension is created, the probability of dehiscence and bleeding or pseudoaneurysm formation is lowered. As a result, operation time is shortened since minimall aortic resection and no coronary ostia dissection is required and time needed for hemostasis is minimized.

A large body of evidence over the past decades demonstrates that for simulataneous replacement of composite graft of the ascending aorta and aortic valve, the original Bentall procedure and its modifications yield satisfactory outcome [6, 12–15]. Studies have shown that inordinate late intraoperative and early postoperative bleeding and the formation of pseudoaneurysm at the suture lines are the main complications associated with the classic Bentall method [4, 14]. It is important to reduce the operation time of the Bentall procedure in order to reach the minimum time for optimum hemostasis. Pevious research have explored various options and modifications in order to improve hemostasis and prevent bleeding. For instance, some studies have investigated the possible effects of Teflon on diminished blood loss during coronary artery anastomosis [16]. Teflon felt amplification in the aortic root has been used in acute type A aortic dissection operation. In this approach, the Teflon felt was only implanted between the dissected layers of the aorta to remodel intimae layers, but was not used as the conservator layer [17]. Miller and Mitchell described the use of a doughnut of Teflon felt or autologous pericardium placed around the coronary ostial aspect of the coronary buttons to prevent tissue tearing [18]. Reinforcement with autologous pericardium during coronary artery anastomosis prevented late pseudoaneurysm; however, pseudoaneurysms of coronary ostia anastomoses were reported at the proximal aortic suture line [18]. Recently, Della Corte et al. have suggested that in order to decrease post-operative bleeding, using imbricated suture-line stitches at the proximal part of vessel and spontaneous subsequent fibrin-sealant spraying were related with low rates of complications, including bleeding, renal and respiratory dysfunctions in short-term follow up with the modified Bentall procedure [19]. Besides, some studies suggested that modified Bentall procedure with a Carrel patch and inclusion technique could enhance hemostasis [15, 20]. The advantages of performing the Bentall procedures with a Carrel patch are that coronary ostial implantation into the prosthetic graft can be well executed with full visualization and that stress on the anastomosis can be averted [15].

An open button technique is the appropriate choice for performing composite conduit replacement of the ascending aorta; however,when insertion of the conduit is completed an excessive post-operative bleeding is still the main problem [10]. Some studies can solve this problem by suturing the coronary ostia in a double layer fashion with an “endo-button” technique [21], which provides a wide adherence surface against the graft and increases hemostasis. In contrast with the Teflon felt method, this technique does not need additional supportive suture by external material and as in cases of dissection, it can also be performed on the fragile aortic wall [22]. One of the remaining issues with the button technique is the injury of epicardium when the coronary buttons are going to be built. Complete coverage by the epicardium serves to concentrate infiltration from the needle hole in the wrapping. To prevent this, the time of hemostasis and bleeding volume should be controlled [15]. In this study, we found no incidence of coronary pseudoaneurysms while some studies indicated that the incidence of a coronary ostial pseudoaneurysm when using a button technique varies from 3.1 to 9 % [4, 13].

Cabrol technique is a safe, non-invasine and affordable technique, having a crucial role in cases of reoperation, severe calcification of the ostia, difficult mobilization of the coronary arteries and extreme aortic dilation [23]. Also, this technique could be used in coronary or aortic valve intervention in presence of chronic regeneration or familial hypercholesterolemia which leads to porcelain aorta [24, 25]. The technique makes the anastomoses of the coronary arteries to the aortic tube when other reimplantation techniques fail to do so. For example, placing the brittle coronary ostia in an individual with thorough dissection is difficult in the button approach, thus using the Cabrol aortocoronary anastomosis is preferred [24, 25]. It seems that the Bental operation and its Button modification have higher levels of mortality than Cabrol. Moreover, Cabrol is still considered a first-line approach in cases with specific conditions. Yet, button modification of the Bentall procedure remains as the standard treatment in approximately all patients [23, 26].

In the current study, the mean duration of cardiopulmonary bypass was 144 ± 48 min and the average postoperative bleeding was 450 ± 105 mL (326 ± 95 mL in the first postoperative day and 130 ± 64 mL in the second day) which both were lesser in comparison with some similar studies [19, 27]. In a modification of the Bentall procedure, Della Corte et al used a combination of imbricated proximal suture-line stitches followed by fibrin-sealant spraying to improve hemostasis. The mean cardiopulmonary bypass time was 166 ± 50 min, which is longer than observed here [19]. Despite efforts to reinforce the sutures and seal the sites of oozing, Della Corte et al reported that the total bleeding volume in the first and second postoperative day amounted to more than 1000 mL, which is significantly higher than the amount of blood loss observed here [19]. Increased cardiopulmonary bypass time have been associated with an increased risk of mortality following composite valvle graft replacement of the aorta [28, 29]. In a retrospective account of 348 patients, Svensson et al observed that the risk of early mortality in patients with cardiopulmonary bypass time exceeding 98 min is five times more than patients undergoing bypass for 65 min or less [29].

In the present series of patients, 5-year survival rate was 97 % . Only ten (9 %) patients needed 3 days or more to stay in the ICU due to hemodynamic or respiratory problems. The mean duration of hospital stay was 5 ± 2 days. On the follow-up, no cases were reported with a thrombosis or perfusion in the space between the aortic tube and the graft. Also, no endocarditis, pericardial effusion and GI bleeding, which were related to the operation were reported.

## Conclusion

In conclusion, the current study found that the modified Bentall procedure provided satisfactory results, including reduced operation and cardiopulmonary bypass time and reduced post-operative bleeding in patients requiring aortic root replacement. Pseudoaneurysms, coronary events and prosthesis-related complications were not observed. Future research should also focus in defining type and intensity of follow-ups and provide anatomic and flow characteristics to enable comparisons between different modifications of the Bentall technique.

### Ethical issue

The study was conducted in accordance with the principles of Declaration of Helsinki 1996 version and Good Practice standards. All subjects signed informed-consent forms.
